# Metabolic syndrome and risk of subclinical hypothyroidism: a systematic review and meta-analysis

**DOI:** 10.3389/fendo.2024.1399236

**Published:** 2024-06-25

**Authors:** Lei Zhong, Shuo Liu, Yao Yang, Tong Xie, Jifeng Liu, Huahui Zhao, Guang Tan

**Affiliations:** ^1^ Department of General Surgery, The First Affiliated Hospital of Dalian Medical University, Dalian, Liaoning, China; ^2^ Department of Endocrinology and Metabolic Diseases, The First Affiliated Hospital of Dalian Medical University, Dalian, Liaoning, China; ^3^ Institute of Integrative Medicine, Dalian Medical University, Dalian, Liaoning, China

**Keywords:** subclinical hypothyroidism, thyroid, metabolic syndrome, metabolic component, meta-analysis

## Abstract

**Background:**

Subclinical hypothyroidism (SCH) is a common endocrine subclinical disorder, the main adverse consequences of which are the development of clinical hypothyroidism and the promotion of ischemic heart disease. Metabolic syndrome (MetS) is a collection of metabolic problems. The goal of this meta-analysis was to evaluate the relationship between MetS and SCH.

**Methods:**

Suitable publications were identified using PubMed, Embase, and the Cochrane Library. The meta-analysis included only studies in English that reported odds ratio (OR) data for MetS and SCH. Two researchers combined data using a random-effects model. OR and 95% confidence intervals (CIs) were used to present the results.

**Results:**

MetS was associated with an elevated risk of developing SCH (OR 2.56, 95% CI 1.44–4.55). However, the individual components of MetS were not associated with the risk of SCH. Subgroup analysis revealed that different definitions of MetS had varying effects on SCH. Sensitivity analysis confirmed that our results were robust.

**Conclusions:**

This meta-analysis indicates that patients with MetS have an increased risk of SCH, while there is no significant association between the five individual components of MetS and the risk of SCH.

**Systematic review registration:**

https://www.crd.york.ac.uk/PROSPERO/, identifier CRD42023454415.

## 1 Introduction

Metabolic syndrome (MetS) is a pathological state of a variety of metabolic disorders, including obesity, hyperglycemia, hypertension, and dyslipidemia (1, 2). It is recognized as one of the clinical syndromes that significantly impact human health (1, 3–6), affecting an estimated 25% of the world’s population (4, 6, 7). The risk of cardiovascular disease would be significantly increased when these metabolic abnormalities co-exist in an individual.

Subclinical hypothyroidism (SCH) is a metabolic disease that has no obvious clinical symptoms and signs, and the thyroid hormone level is normal and the thyroid-stimulating hormone (TSH) in the blood is elevated (8, 9). A growing body of research shows that SCH is associated with lipid abnormalities, increased cardiovascular risk, and metabolic disorders such as high blood pressure, chronic inflammation, and a hypercoagulable state of the blood, especially in older women (10). Many studies have also pointed to thyroid disorders as being complications of MetS and type 2 diabetes (11, 12). Numerous studies have shown a connection between thyroid hormone and TSH levels in serum and elements of the MetS (13–16). For instance, in a study by Kim et al., serum free thyroxine 4 (FT4) concentrations were found to be positively correlated with blood pressure, fasting glucose, high-density lipoprotein cholesterol (HDL-C), and triglyceride (TG) levels (13). Furthermore, MetS and SCH have been frequently linked in the studies (17). In a study of 2,119 people aged 70 to 79 years, Antika et al. found that elevated TSH levels increased the risk of MetS (1). However, the relationship between MetS and its five components with SCH remains a subject of debate (18, 19). Consequently, we conducted this systematic review and meta-analysis to explore whether MetS and its components are associated with an increased risk of SCH.

## 2 Method

The study has been reported according to PRISMA (Preferred Reporting Items for Systematic Reviews and Meta-Analyses), and the registration number is CRD42023454415 (PROSPERO registration platform).

### 2.1 Search strategy

In the PubMed, Cochrane Library, and EMBASE databases, two researchers (SL and LZ) independently looked for publications published from January 1988 to March 2023. The following were searched for words: (“Subclinical hypothyroidism” OR “SCH” OR “hyperthyroidism” OR “sub-clinical thyroid deficiency”) AND (“Metabolic Syndrome” OR “Metabolic Cardiovascular” OR “Dysmetabolic” OR “Metabolic X”). We only considered research that was written in English. To find prospective acceptable articles, we also looked for and read the complete contents of references from the original studies.

### 2.2 Selection criteria

The following were the research’s inclusion requirements: (1) observational studies (cohort studies, case–control studies, and cross-sectional studies) published in English; (2) the primary outcome was the effect of MetS on SCH prevalence; and (3) there were sufficient data to do a comprehensive analysis. Studies, however, were instantly disqualified when they met any of the following requirements: (1) publications are studies such as case reports, animal experiments, or conference abstracts that do not provide critical data; and (2) diagnostic criteria for MetS and SCH were not provided.

### 2.3 Data extraction

The following information was gathered by SL and LZ: initially, the basics (first author’s name, publication year, and place of publishing); second, participant data (size of the sample, mean age, and sex ratio); and third, MetS and SCH diagnostic standards. Two examiners independently extracted the data and cross-checked them and individually evaluated the quality of every research according to the Newcastle–Ottawa Scale (NOS) and the Agency for Healthcare Research and Quality (AHRQ). Every study was given a rating based on whether it was low (<4), medium, or high quality (>8). All disagreements were worked out by mutual consent and discussion with another author (JFL).

### 2.4 Statistical analyses

Odds ratios (ORs) and its 95% CI were used to assess the correlation between MetS and SCH. Furthermore, we evaluated the effects of each MetS factor on the risk of SCH. The *I* ^2^ was applied to evaluate statistical heterogeneity for each study. An *I* ^2^ statistic of less than 25% indicates low heterogeneity, a score greater than 75% indicates high heterogeneity, and a score between the two indicates moderate heterogeneity. When the heterogeneity of the study is large, subgroup analysis and/or meta-regression will be used to find the source of heterogeneity. Sensitivity analysis was used to measure the stability of the study. All data were combined using a random-effects model. Publication bias was evaluated through Egger’s test and Begg’s test. All analyses were performed by Stata, and *p* < 0.05 denotes statistical significance.

## 3 Results

### 3.1 Search results

**Figure 1:**
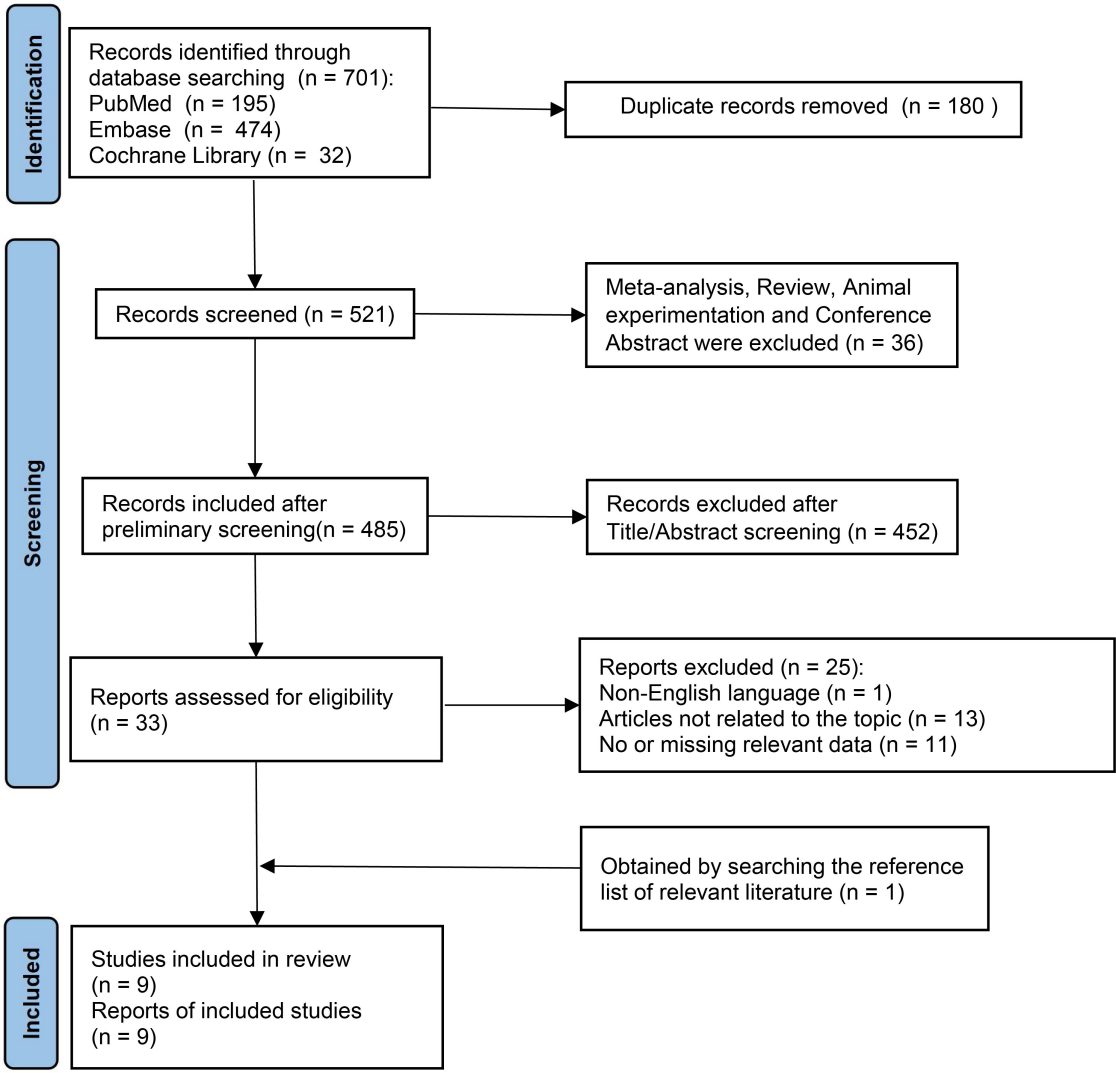
Flowchart of selecting studies.

A flowchart that depicts the literature screening procedure is shown in **Figure 1**. A total of 701 studies were found in the database. Studies that failed to meet the inclusion criteria and all duplicate articles were removed. Nine studies (2, 18–25) in total met the inclusion requirements for the present analyses. On the chosen literature, we performed a meta-analysis and systematic review.

### 3.2 Characteristics of the included studies

The study comprises research published between 2007 and 2022, and it received an average quality rating of 8.7 stars (**Supplementary Tables S1**–**S3**). There were three different kinds of investigations, including a cohort study, four case–control studies, and four cross-sectional studies. **Table 1** provides a summary of the fundamental characteristics of each study included in this meta-analysis.

**Table 1:** Characteristics of the studies included in the quantitative and qualitative review.

Author/Publication year	Study design	Region	Mean age	Sample size	Male/Female	MetS diagnosis criteria	Diagnostic criteria for subclinical hypothyroidism	Study quality
Uzunlulu 2007	Case–control	Turkey	47.48 ± 11.62	410	101/309	NCEP-ATP III	–	8
Meher 2013	Case–control	India	47.02 ± 5.06	150	69/81	NCEP-ATP III	10 µIU/mL > TSH > 4.20 µIU/mL with normal FT4 (0.27–4.2 µIU/mL) and FT3 (1.4–4.2 pg/mL)	8
Udenze 2014	Cross-sectional	Nigeria	48.43 ± 11.09	150	105/68	NCEP-ATP III	–	8
Gyawali 2015	Cross-sectional	Nepal	MetS group: 30–70 Control group: 26–70	699	–	NCEP-ATP III	TSH > 4.20 µIU/mL with normal FT4 and FT3	9
Chang 2017	Cohort study	China	–	66,822	–	AHA	TSH > 5 µU/mL with normal FT4 (4.5–12 µg/dL)	9
Saluja 2018	Case–control	India	52.7 ± 14.14	200	86/114	IDF	–	9
Suhashini 2018	Case–control	India	35–55	90	–	NCEP-ATP III	–	8
Mehran 2021	Cross-sectional	Iran	40.4 ± 14.2	4,905	2,152/2,753	AHA	TSH > 5.06 µ/mL with normal FT4 (0.91–1.55 ng/dL)	10
Rao 2022	Cross-sectional	India	20–75	150	–	NCEP-ATP III	–	9

SCH, subclinical hypothyroidism; MetS, metabolic syndrome; TSH, thyroid-stimulating hormone; FT4, free tetraiodothyronine; FT3, free triiodothyronine; NCEP-ATP III, National Cholesterol Education Program-Adult Treatment Panel III; IDF, International Diabetes Federation; AHA, American Heart Association/National Heart, Lung, and Blood Institute; -, Not provided in the study.

### 3.3 Meta-analysis results

**Figure 2:**
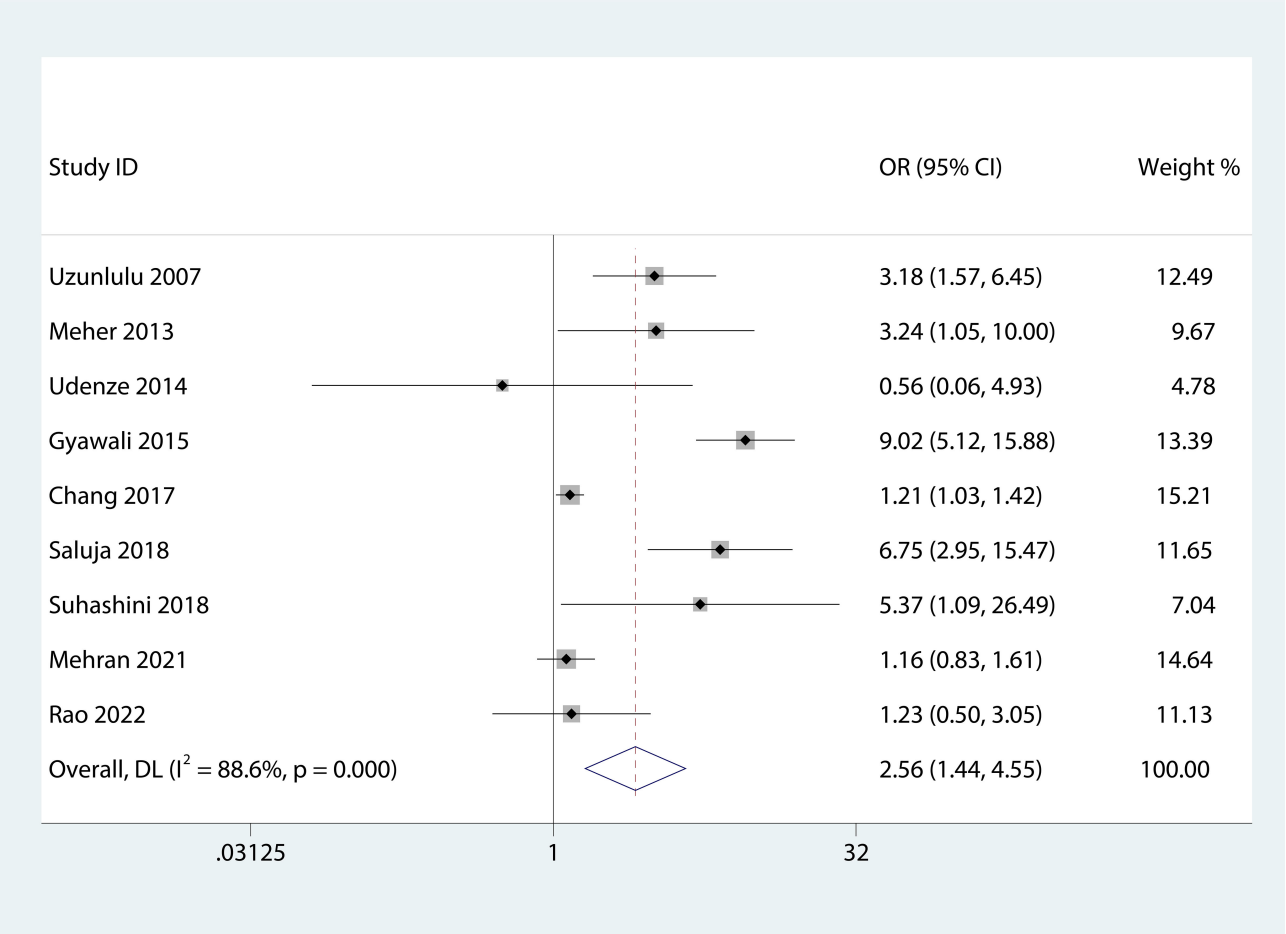
Forest plot of the relationship between MetS and SCH.

**Figure 3:**
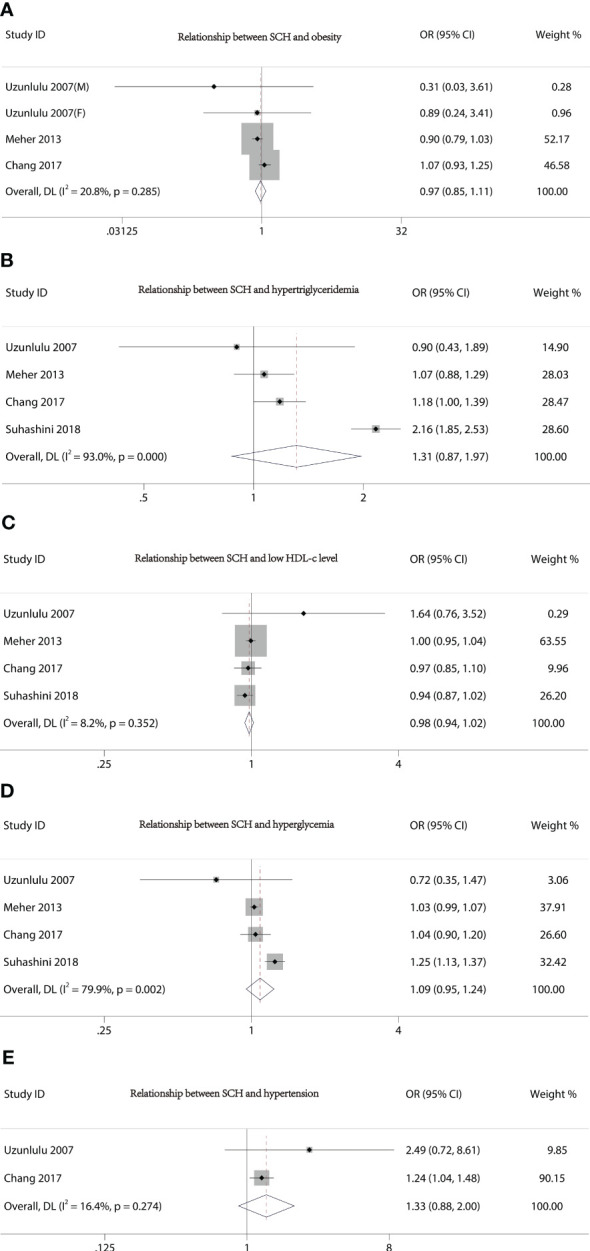
Forest plot of the relationship between MetS components and SCH. **(A)** Relationship between SCH and obesity. **(B)** Relationship between SCH and hypertriglyceridemia. **(C)** Relationship between SCH and low HDL-c level. **(D)** Relationship between SCH and hyperglycemia. **(E)** Relationship between SCH and hypertension.

**Figure 2** presents the forest plots from the meta-analysis of SCH and MetS. In contrast to non-MetS patients, those with MetS had a higher prevalence of SCH (OR 2.56, 95% CI 1.44–4.55). However, our study did not find an association between the various components of MetS and the incidence of SCH (**Figure 3**).

### 3.4 Sensitivity analysis and publication bias

**Figure 4:**
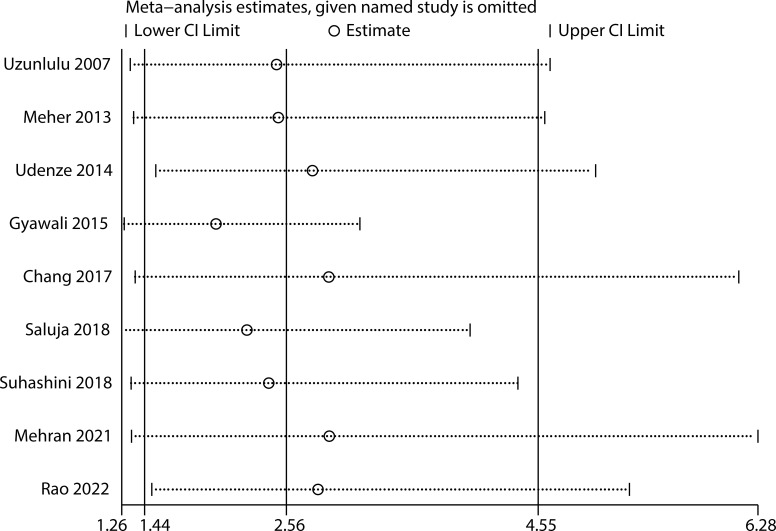
Sensitivity analysis of studies on the effects of MetS on SCH.

**Figure 5:**
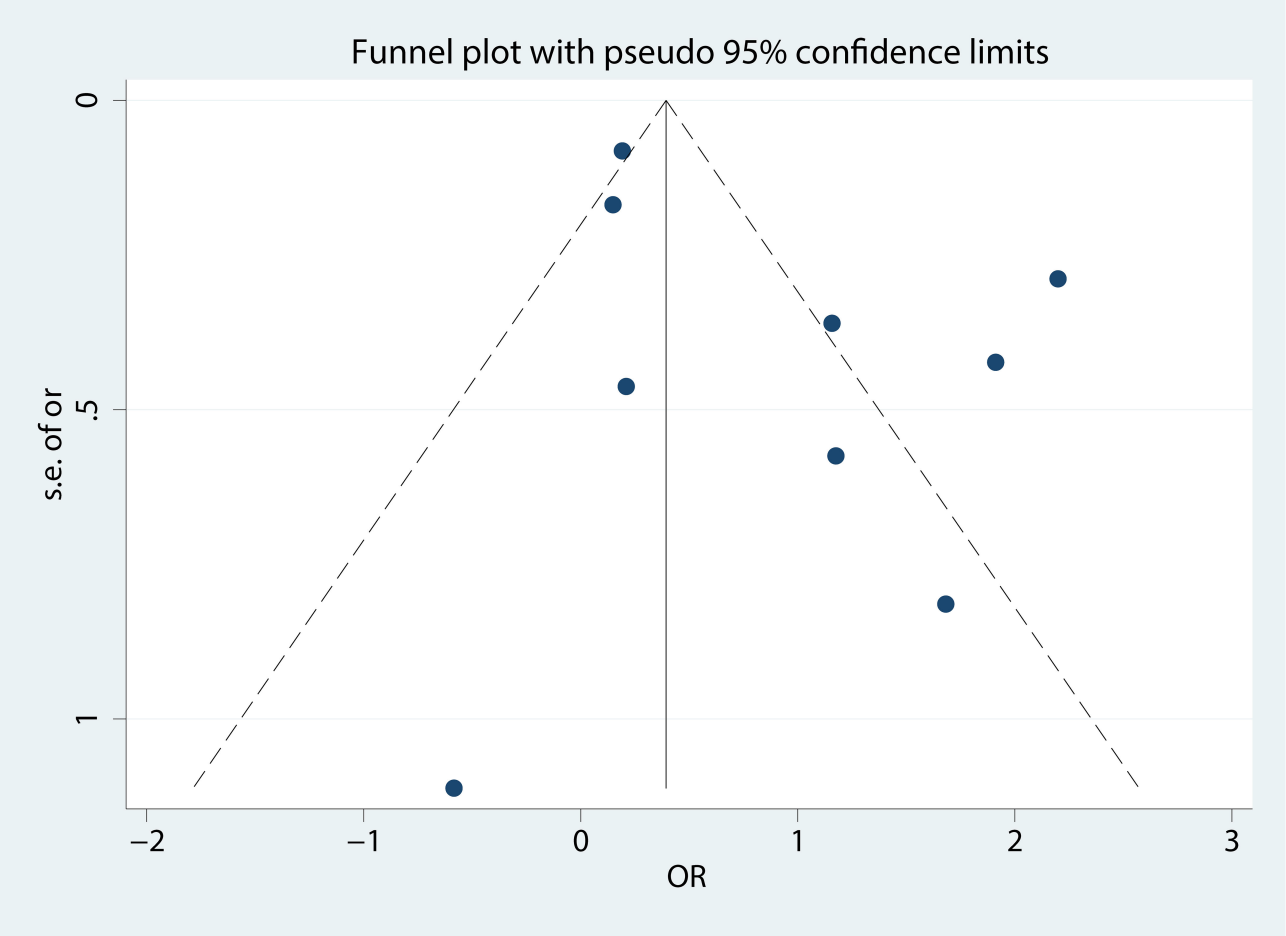
Funnel plot of studies on the effects of MetS on SCH.

Sensitivity analyses of nine articles showed that arbitrary deletion of the literature in this study will not affect the results of this study, meaning that the above results are stable and reliable (**Figure 4**). Either Egger regression analysis or funnel plots (**Figure 5**) indicated the presence of no publication bias for our analyses.

## 4 Discussion

Obesity, hypertension, hyperlipidemia, and hyperglycemia are among the metabolic risk factors that can occur together to form MetS. It is significant to note that multiple cross-sectional studies revealed a connection between SCH and MetS and its components (26–28). Our meta-analysis comprehensively assessed the association between SCH and MetS by taking into account and evaluating the results of nine independent observational studies. Our results were in agreement with the majority of previous studies in that MetS would increase the risk of developing SCH. Surprisingly, our study found no significant association between the individual components of MetS and the risk of SCH. Analysis of sensitivity and the detection of publication bias supported the stability of our findings.

Although hypothyroidism is frequently thought to be secondary to weight gain (7), more recent arguments have been made that hypothyroidism may be secondary to obesity (26, 29–31). A possible explanation was that leptin, cytokines, and other inflammatory markers are produced by excessive adipose tissue (31), which may inhibit sodium/iodide symporter mRNA expression and disrupt iodide uptake activity in thyroid cells (32, 33) or modulate the expression and activity of deiodinases (34, 35). Additionally, evidence from people and a mouse model suggests that obesity causes fat to build up in the thyroid gland. Studies on obese mice suggest that this may have an impact on the thyroid’s ability to produce hormones and cause SCH (36). The cause of the link between fat and hypothyroidism still has to be clarified, though.

Numerous studies have revealed that people with diabetes may have different serum concentrations of thyroid hormones (37). Type 2 diabetes mellitus has been proven to be negatively correlated with serum TSH38 levels (37), and it has been shown that poorly controlled diabetes removed the nocturnal TSH peak because the TSH response to TRH was disturbed (38). In a few studies, it has also been shown that SCH leads to insulin resistance (39, 40). This connection between hypothyroidism and insulin resistance, which refers to one such scenario where insulin resistance plays a key role in the clustering of risk factors for cardiovascular disease, can help to explain why people with MetS experience an elevated incidence of hypothyroidism.

Although it is generally accepted that there is a strong relationship between hypercholesterolemia and clinical hypothyroidism (41), Chang et al.’s analysis suggests that high serum triglycerides may be an important independent factor in increasing SCH risk (18). Meanwhile, Shao and colleagues also discovered that rats fed a high-fat lard diet for 24 weeks had significantly higher serum triglyceride levels in both the serum and thyroid tissue, lower serum total and free T4 levels in conjunction with higher serum TSH levels, and altered macro- and micromorphology of the thyroid gland (42). Furthermore, Han and colleagues showed in a study on animals that a high-fat diet could harm mice’s thyroid glands and result in a thyroid hormone disorder (43). In our investigation, no correlation between hypertriglyceridemia and SCH was found. It is necessary to conduct more research to determine whether dietary factors may play a role in SCH incidence in those who are at risk.

In a recent study, Cai et al. (44) examined the connection between thyroid function and various forms of hypertension. They discovered that patients with clinical hypertension had higher serum TSH levels than patients with clinical normal blood pressure, and that people with ambulatory hypertension frequently had higher serum TSH levels than people with ambulatory normal blood pressure (44). Another study carried out in India revealed that individuals with high blood pressure had much higher average TSH than the general population (45). In addition, the study found that in new cases of hypothyroidism across all cohorts, SCH was more common than overt hypothyroidism (45). This is due to the fact that SCH is indicative of the early or beginning phases of thyroid illness, which, if left untreated, can result in severe hypothyroidism (45). Furthermore, among all the pathogenic pathways that might result in hypertension, a number of them are linked to hypothyroidism (46). These pathways include altered catecholamine levels in the blood, perturbations to the renin–angiotensin–aldosterone system, and elevated peripheral vascular resistance (47, 48). Therefore, we believe that there may be an interactive relationship between hypertension and SCH. However, since most of these studies are cross-sectional studies, more clinical trials and basic research are needed to confirm them.

Furthermore, there was strong heterogeneity in the results of our study. The reasons for this may be the differences in the diagnostic criteria of MetS and SCH, study population, and epidemiological study methods included in the study. Further prospective, multicenter, large cohort studies are needed to confirm this.

Additionally, the study contains some flaws. First of all, since the majority of the literature used in this investigation was observational, it might be challenging to differentiate between cause and effect from the correlation between SCH and MetS. Secondly, there have not been as many investigations on the connection between MetS and SCH, which might make the findings less trustworthy. Thirdly, the results of our study have strong heterogeneity, and we were unable to conduct subgroup analyses for additional characteristics, such as sex and age, due to a lack of data, which prohibited us from exploring the potential relationship between MetS and SCH in greater detail. Moreover, most of the articles selected for our study were based on people from China and India, which may have influenced the results. Therefore, it is necessary to conduct more longitudinal large-scale prospective cohort studies to determine whether MetS may play a role in SCH incidence.

In our meta-analysis of nine studies, the patients with MetS were found to be associated with an increased incidence of SCH. However, no significant association was found between the five components of MetS and the risk of SCH.

## Data availability statement

The original contributions presented in the study are included in the article/**Supplementary Material**. Further inquiries can be directed to the corresponding authors.

## Author contributions

LZ: Conceptualization, Data curation, Investigation, Writing – original draft, Writing – review & editing. SL: Conceptualization, Methodology, Software, Writing – original draft, Writing – review & editing. YY: Writing – original draft, Data curation, Validation, Investigation. TX: Writing – original draft, Data curation, Validation, Investigation. JL: Conceptualization, Investigation, Methodology, Writing – review & editing. HZ: Conceptualization, Methodology, Writing – review & editing. GT: Conceptualization, Writing – review & editing.

## Supplementary material

Supplementary Figure 1 Different MetS diagnosis criteria.
