# Supplementary figures and images for: Metabolic syndrome and risk of subclinical hypothyroidism: a systematic review and meta-analysis
Source: Front Endocrinol (Lausanne). 2024 Jun 25;15:1399236. doi: 10.3389/fendo.2024.1399236 (PMC11231392; doi:10.3389/fendo.2024.1399236)

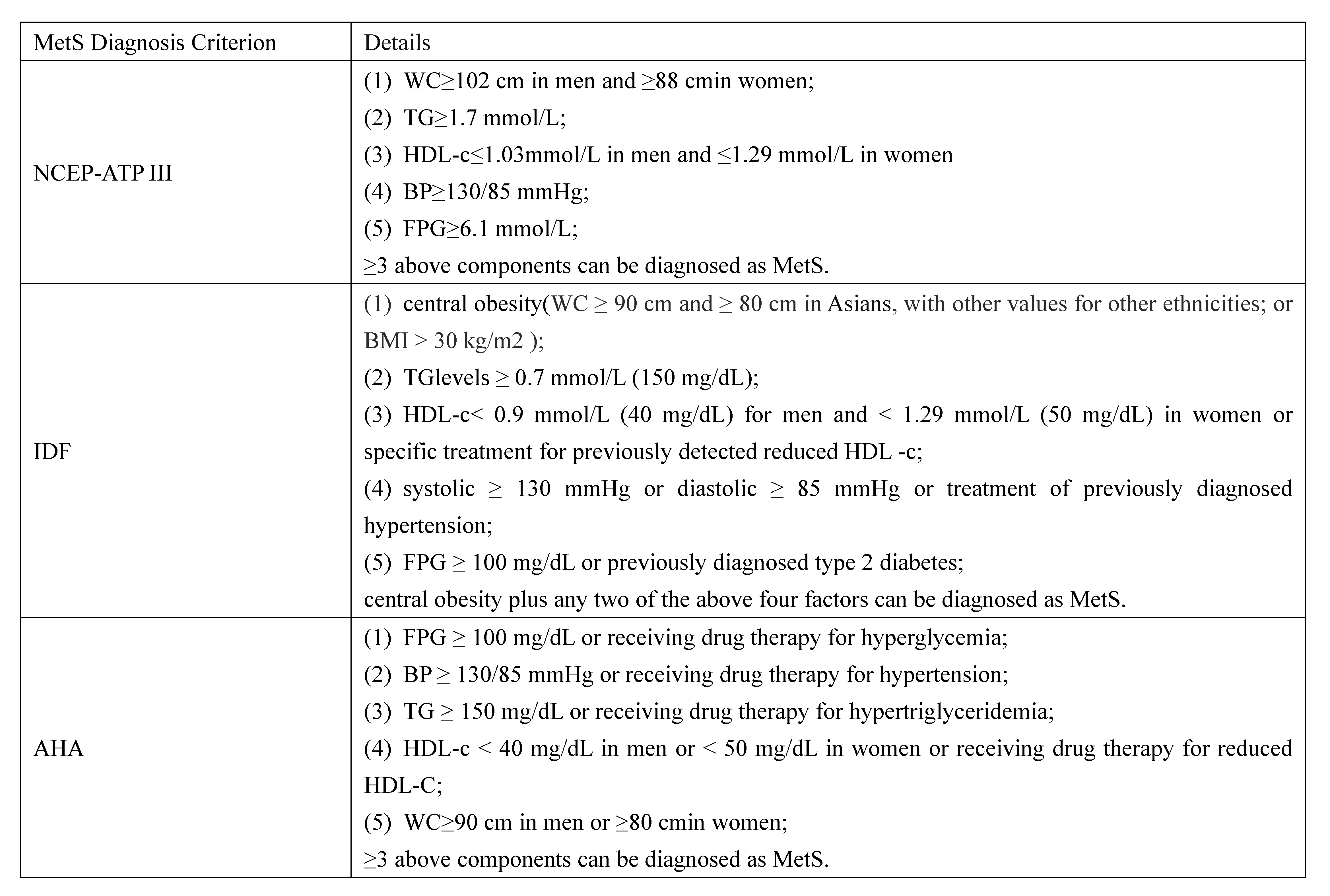

Supplement: Supplementary Figure 1 — Different MetS diagnosis criteria. [file Image_1.tif]
