# Supplementary material for: Metabolic syndrome and risk of subclinical hypothyroidism: a systematic review and meta-analysis
Source: Front Endocrinol (Lausanne). 2024 Jun 25;15:1399236. doi: 10.3389/fendo.2024.1399236 (PMC11231392; doi:10.3389/fendo.2024.1399236)
Supplement: Supplementary file 2 [file DataSheet_1.docx]

# Table S1. Results of quality assessment for the included cross-sectional studies.

| Agency for Healthcare Research and Quality (AHRQ)  Item | Udenze 2014 | | | Gyawali 2015 | | | Mehran 2021 | | | Rao 2022 | | |
| --- | --- | --- | --- | --- | --- | --- | --- | --- | --- | --- | --- | --- |
|  | Y | N | U | Y | N | U | Y | N | U | Y | N | U |
| 1) Define the source of information (survey, record review) | ★ |  |  | ★ |  |  | ★ |  |  | ★ |  |  |
| 2) List inclusion and exclusion criteria for exposed and unexposed subjects (cases and controls) or refer to previous publications | ★ |  |  | ★ |  |  | ★ |  |  | ★ |  |  |
| 3) Indicate time period used for identifying patients |  | ★ |  | ★ |  |  | ★ |  |  | ★ |  |  |
| 4) Indicate whether or not subjects were consecutive if not population-based | ★ |  |  | ★ |  |  | ★ |  |  | ★ |  |  |
| 5) Indicate if evaluators of subjective components of study were masked to other aspects of the status of the participants |  | ★ |  |  | ★ |  | ★ |  |  |  | ★ |  |
| 6) Describe any assessments undertaken for quality assurance purposes (e.g, test/retest of primary outcome measurements) | ★ |  |  |  |  | ★ | ★ |  |  | ★ |  |  |
| 7) Explain any patient exclusions from analysis | ★ |  |  | ★ |  |  | ★ |  |  | ★ |  |  |
| 8) Describe how confounding was assessed and/or controlled | ★ |  |  | ★ |  |  | ★ |  |  | ★ |  |  |
| 9) If applicable, explain how missing data were handled in the analysis | ★ |  |  | ★ |  |  | ★ |  |  | ★ |  |  |
| 10) Summarize patient response rates and completeness of data collection | ★ |  |  | ★ |  |  | ★ |  |  | ★ |  |  |
| 11) Clarify what follow-up, if any, was expected and the percentage of patients for which incomplete data or follow-up was obtained |  | ★ |  | ★ |  |  |  |  | ★ |  | ★ |  |
| ***Quality scores*** | 8 | | | 9 | | | 10 | | | 9 | | |

Y, Yes; N, No; U, Unclear; an item would be scored ‘0’ if it was answered ‘NO’ or ‘UNCLEAR’; if it was answered ‘YES’, then the item scored ‘1’ (Question 5 take reverse scoring).

# Table S2. Results of quality assessment for the included case-control studies

|  |  | Uzunlulu 2007 | Meher 2013 | Saluja 2018 | Suhashini 2018 |
| --- | --- | --- | --- | --- | --- |
| ***Selection*** | Adequate definition of cases | ★ | ★ | ★ | ★ |
|  | Representativeness of the cases | ★ | ★ | ★ | ★ |
|  | Selection of controls | ★ | ★ | ★ | ★ |
|  | Definition of controls | ★ | ★ | ★ | ★ |
| ***Comparability Control for important factors*** | | ★★ | ★★ | ★★ | ★★ |
| ***Exposure*** | Ascertainment of exposure | ★ | ★ | ★ | ★ |
|  | Same method of ascertainment for cases and controls | ★ | ★ | ★ | ★ |
|  | Non-response rate | - | - | ★ | ★ |
| ***Quality scores*** | | 8 | 8 | 9 | 8 |

# Table S3. Results of quality assessment for the included cohort study.

|  |  | Chang 2017 |
| --- | --- | --- |
| ***Selection*** | Representation of the exposed cohort | ★ |
|  | Selection of non-exposed cohort | ★ |
|  | Ascertainment of exposure | ★ |
|  | None of the subjects had already developed the disease at the start of the study | ★ |
| ***Comparability of exposed and non-exposed cohorts*** | | ★★ |
| ***Outcome*** | Assessment of outcome | ★ |
|  | Whether follow-up is long enough for the disease under study | ★ |
|  | Adequacy of follow-up of cohorts | ★ |
| ***Quality scores*** | | 9 |
